# Refractory tenosynovitis with ‘rice bodies’ in the hand due to *Mycobacterium intracellulare*

**DOI:** 10.1007/s15010-015-0844-0

**Published:** 2015-09-26

**Authors:** Ho Namkoong, Keizo Fukumoto, Igen Hongo, Naoki Hasegawa

**Affiliations:** Division of Pulmonary Medicine, Department of Medicine, Keio University School of Medicine, Shinjuku, Tokyo Japan; Saitama Hand Surgery Institute of Saitama Seikeikai Hospital, Higashimatsuyama, Saitama Japan; Division of Infectious Diseases, Musashino Red Cross Hospital, Musashino, Tokyo Japan; Center for Infectious Disease and Infection Control, Keio University School of Medicine, Shinjuku, Tokyo, 160-8582 Japan

## Legend

**Fig. 1:**
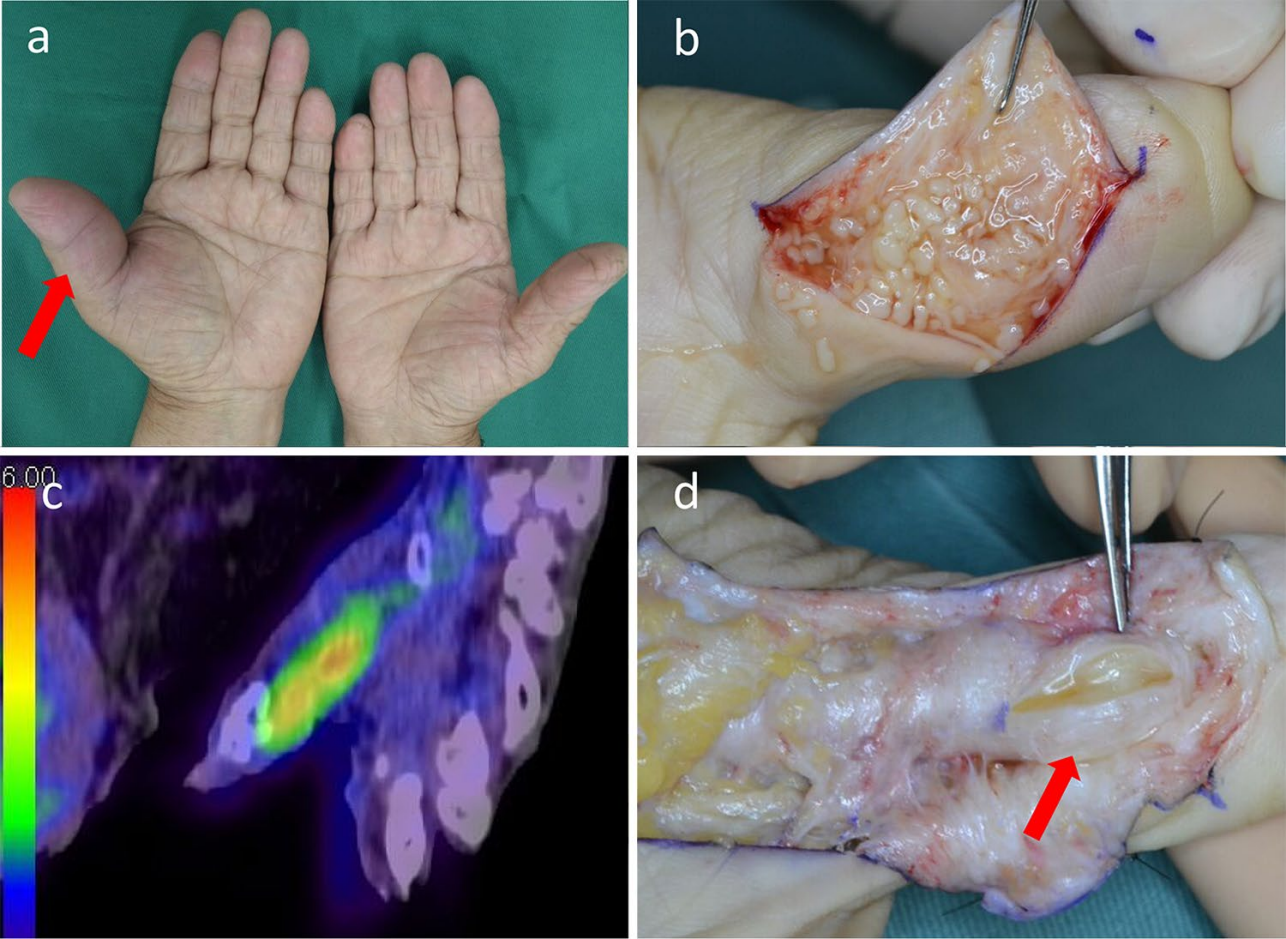
**a** Swelling and tenderness of the left thumb and thenar before the first operation (*red* *arrow*). **b** ‘Rice bodies’ were observed during the first open drainage performed for treating tenosynovitis. **c** ^18^F-fluorodeoxyglucose positron emission tomography/computed tomography showed intense uptake around the metacarpophalangeal joint, before the third drainage. **d** Intraoperative findings at the third drainage showed synovial thickening and synovial fluid retention. By this time, the ‘rice bodies’ had resolved (*red* *arrow*)

A 76-year-old man presented with a 2-month history of swelling and tenderness of the left thumb and thenar. The patient had type 2 diabetes mellitus. He had been bitten by a dog on the left hand 1 year previously, and the wound had healed without treatment. He was initially diagnosed with non-infectious tenosynovitis and received steroid injections repeatedly (Fig. 1a). Thereafter, open diagnostic-drainage was performed, and the presence of ‘rice bodies’ was visually noted in the hand (Fig. 1b). Based on the pathological finding of granuloma and positive specimen culture for *Mycobacterium intracellulare,* he was diagnosed with tenosynovitis due to *Mycobacterium avium* complex (MAC). While his symptoms initially improved by isoniazid, rifampicin, and ethambutol, the redness and tenderness around the left wrist gradually worsened at 6 months after the first operation. Then therapeutic-drainage was performed again, and the regimen was changed to clarithromycin, rifampicin, ethambutol, and sitafloxacin after the introduction to our department. After 1 year, however, a nodule developed around the metacarpophalangeal joint, associated with an intense uptake on ^18^F-fluorodeoxyglucose positron emission tomography/computed tomography (Fig. 1c), implying the residual inflammation. Therapeutic-drainage was performed again (Fig. 1d), and he is now in remission under antimicrobial chemotherapy.

MAC tenosynovitis is a refractory infectious disease, reported more commonly in Asians [1–3]. Most patients have an injury history and often require multiple operations as in this case [1–4]. The presence of ‘rice bodies’ is a characteristic intraoperative finding as well as tuberculosis [3, 5]. Although the appropriate duration of chemotherapy is unclear, past studies recommended a 1–2-year treatment period [4]. When seeing cases present with refractory tenosynovitis, MAC tenosynovitis should be considered in the differential diagnosis.
